# Porcelain as a Beneficial Art in Dentistry

**Published:** 1900-11-15

**Authors:** F. J. Capon

**Affiliations:** Toronto, Canada


### PORCELAIN AS A BENEFICIAL ART IN DENTISTRY.

BY F. J. CAPON, D.D.S., M.D.S., TORONTO, CANADA.

(Read before the Dental Society of the State of New York, May 9, 1900, and reprinted from the *Dental Cosmos*.)

"The true work of art," says Michael Angelo, "is but a shadow of the divine perfection."

"The art of an age or nation," writes another, "is the efflorescence of its whole spiritual life and endeavor." The art of a people is therefore only the embodiment of its prevailing ideas, affections and conceptions in architecture, in sculpture, and on the glowing canvas.

Thus a dentist may be skilled in mechanism, but still lack the innate art which is required to produce nature's conceptions. A porcelain worker requires at least a percentage of this innate art. Unless he is born with faculties that he can cultivate, and which will enable him to become proficient; unless he is born with talents that will develop a fine artistic and mechanical skill, he will never be an expert in porcelain work, though his manual training may be the best the office or college can give; he must have a base to build it on, and that he gets at his birth.

Porcelain was introduced into dentistry about the beginning of the present century. The attempts made were but poor as to form and color, representing the natural organs very imperfectly. Little improvement was made until Dr. Wildman, of Philadelphia, in 1838, produced such results in life-like appearance as had not been obtained before. Thus the progress which has been made in porcelain is credited to the profession of this country, the cradle of all modern dentistry. The introduction of porcelain into dentistry gives a widespread field of art and manipulation, varying in application, yet dovetailing into each other, making it necessary for a student to acquaint himself with the underlying principles of art as well as to have a knowledge of the manufacture of bodies and enamels, with the modes of vitrifying them, whether by gas, electricity, oil or coke.

Much has been said of late as to the merits of high and low grade bodies; possibly they would be more properly classified as high, medium and low. The true high grade is termed block body, which is used by the manufacturers to produce the gum blocks and all artificial teeth which are the nearest production we have to the natural appearance. Thus as we vary in grade from the block bodies we lose the transparent, life-like appearance.

The medium are the bodies put on the market as highgrade. They work fairly well in producing shade, contour and strength.

The low body, as found on the market, should hardly be classified as a body, as it is composed of such a large proportion of glass as to render it useless for high grade work in the furnace, and as a substitute is a failure, producing poor results which discourage the beginner, "porcelain" being set aside in disrepute and the attempts dubbed as failures, when an expert could have done but little better with the same materials.

I do not wish to appear presumptuous in classifying the bodies; perhaps it is a matter of personal experience; but I have bought the different bodies, good, bad and indifferent, as they were put on the market, until I have "bodies to burn," nevertheless I find that I always set aside the lower and return to the higher. The question of the grades of bodies has been thoroughly threshed; it has come up from time to time in convention, and can only be settled to the satisfaction of each by his trying for himself.

I have seen many dentists fusing the porcelain body by the watch, which explains many failures. This rather amuses the expert, as porcelain, like dough, can be overbaked. It should be governed by the eye, as the different conditions, if only slight, would change the time of fusing. The pressure of gas is not always uniform, and thus the fusing point in the furnace varies. The size of the piece being fused must also be taken into consideration, especially if electricity is employed.

The furnace is a matter of mere choice, as a high class one should be able to bake the higher grade bodies as well as the lower, and *vice versa*, it being only a matter of degrees of heat. I mention gas, electric and oil as the furnaces to choose from; having each in my laboratory, I can speak with more assurance as to their merits. Although the old coke furnace has done and does do its work, it has become obsolete for minor operations; besides it makes the climate too hot for the "white man." Of the three furnaces mentioned, personally I could best dispense with the electric, although it is the one I should advise a beginner to obtain, as having the advantage to him of being sure in fusing without gassing; besides, it is clean and noiseless, and throws out little heat. Its principle objection is the loss of time in fusing and the liability of the wires burning out, which are not quickly or easily repaired.

The oil furnace is a novel invention of Dr. Land, of Detroit. It is designed more particularly to fuse the larger pieces, such as continuous gum work, giving reliable and perfect results, as will be seen by these samples. For those who are not acquainted with it, the diagram will show the scientific manner by which the inventor produced such a tremendous heat by a simple method—the natural draft of a flue.

The gas furnace in my laboratory is also one of Dr. Land's, Midget size, which has been in daily use since 1890 without change or repair, and bids well to be so another decade. It contains a seamless iridium muffle, which has given perfect satisfaction; whereas if the thinner platinum ones are used, one is in constant trouble from either burning through or gassing. Yet, if there is perfect combustion of the gas and air mixture, there is little chance of gassing; a practised eye and ear will at once determine the fact of perfect combustion following the lighting after the turning on of the air pressure pump. The heat will be up and porcelain fused in less than three minutes. This class of furnace may have an objection in the noise that accompanies it; therefore its place is in the laboratory, although it is very entertaining to patients, who often ask to be shown the *modus operandi*.

In summing up: Any furnace will be satisfactory, be it electric or gas, oil or coke, if the operator becomes acquainted with it before he throws it out in disgust. To me the furnace is a part of the laboratory, just as much as the vulcanizer; and if I could carry my wish I would take it in preference, for what class of artificial dentures compares in the great majority of cases to continuous gum? It gives the artistic operator a chance to display his knowledge of art in the reproduction of nature in both the teeth and the gums.

The most important use of porcelain and the furnace in the dental laboratory is in the art of crown work. It is a well known fact that an imitation always suffers by contrast with the original. This, I am forced to say, is too often what happens in replacing artificial crowns. Too many operators there are, endowed with only a fair amount of mechanical skill, who by continued practice are able to place upon a root an apology for a crown, which, as the Dutchman would say, is "good for strong," but that is all the credit due to it. There is no reason in my mind why, when replacing a crown, one can not conform more closely to the unison of nature, combining practical utility with the esthetic. An anatomical crown or cap is not even aspired to by many, they being satisfied to cover the root, say of a molar, with gold without pretense to contour to the adjacent teeth, leaving a condition that is a nuisance to the wearer.

Porcelain comes to the rescue as an ideal method of replacement of the lost crowns of teeth in conspicuous places. It having the required strength, the operator can restore the desired effect according to his ability as an artist.

The advantage of porcelain crowns (made by an operator) over those of gold, or even those combined with gold and porcelain, is obvious. Take, for instance, the ordinary every-day post crown. After the face of the root has been trimmed to the proper shape, an iridium post is fitted into the enlarged canal, the depth of the canal marked on the post, and a disk of pure soft platinum, No. 35, slightly larger than the face of the root is punched in the center, the post forced through to the mark, and soldered with pure gold. This is inserted into the root canal, and the disk burnished and trimmed and burnished again, leaving a margin on the palatine half to form a band. Now, this absolutely fits the face of the root, which is next to an impossibility with a made-up porcelain crown, such as the Logan, etc. And I also claim that a porcelain crown made in this way has an advantage over similarly constructed crowns made with gold backing, in that one is able to more closely imitate nature in shade, position and shape. How difficult to make a natural lapping incisor with a gold backing! Then, again, the shade is often disappointing, as the backing makes it a matter of speculation. But with a porcelain crown the shade can be changed by the aid of mineral paints and burnt in, thus bringing about an exact shade, sometimes impossible to get in a ready made facing. Time is a consideration to most of us, as time saved is money to the dentist; and, after all, this is what we are after. So time is saved in making your own porcelain crown, as one can be made in less time than it takes to go down town to get one of the Logans.

I must speak for the strength of the porcelain crowns, as I think it is an erroneous impression that they are delicate and need protection. I have watched them take the strain of mastication for many years. It seems to me they are stronger than the porcelain facing with gold backing. In the one case the facing is etched to the solid porcelain body of the tooth, practically making the facing and body one; whereas in the other the facing is relying on two little pins, which in soldering are very frequently invisibly checked around their heads, and are liable to be easily forced off if not protected by unsightly gold tips, which are by no means artistic.

Perhaps one of the most satisfactory porcelain crowns is that we call the "jacket" crown. It is especially adapted to broken down incisors and bicuspids where the pulp is intact, and most especially in covering the "peg-shaped" laterals. These "jackets" have proved to be very durable crowns, and have the advantage of enabling one in case of death of the pulp to enter the pulp-chamber without removing or injuring the crown. The record of this crown has, in the past twelve years of my practice, won for it a place that no other single crown can occupy. It has come to my rescue in roots fractured far under the gum (broken by hockey, lacrosse and football); roots badly decayed and under the gum, and most particularly has it been my friend in the wholesale crowning of teeth where it is necessary for the salvation of the pulps.

A great many porcelain workers in making a crown connect the pins of the invested facing with solder to the dowel or some other part. This to me is entirely unnecessary, and I can not see the object of doing it. In fact the pins are a detriment, as a facing with pins will fracture (with the shrinkage of the body or in the heating and cooling) just across the pins. Veneer facings are manufactured for this work, which save time and the trouble of having to entirely grind out the pins to form a proper veneer, and which seldom give trouble in checking. Again, many operators seem to be afraid of grinding the labial surfaces of crowns for fear of destroying the lustrous surface of the facing. That is just what I often like to do; I grind and mutilate (so to speak) until I have obtained the desired shape. It is then made smooth with fine sandpaper and put on a buff of cotton batting, using pumice first, then whiting, which brings a gloss more in keeping with the adjoining natural teeth. If the highest grade bodies are used the gloss can be reproduced by subjecting to high heat in the furnace, but where the medium or low grade bodies are used you would destroy the contour of the crown before the gloss could be obtained, as all vitreous masses under high heat take the spherical form. The color would also be gone and appear like translucent glass.

The use of porcelain for inlays seems recently to be attracting unusual interest in the profession. For the past eleven years I have been working and experimenting with this branch of porcelain art, keeping a close record of successes and failures—I am sorry to say too many of the latter, for nothing would please me more than to find that the "ideal" was porcelain. I have listened to essayists pouring forth the virtues of porcelain, placing it on a pinnacle as the ideal filling, "giving infallible results and absolutely perfect in every detail." I have listened to and have read their sweeping testimonials in favor of porcelain inlays, and felt that they were doing an injustice to their patients, to themselves, to their listeners, and to the system of inlaying. Two or three years' experience may make a man satisfied with himself as an expert, but it does not make porcelain the ideal filling until it meets all the requirements of a true standard and the universal confirmation of the profession.

I do not wish to give an unfavorable impression of porcelain inlays, for they have a place, but the instances of their use are fewer in my practice than ten years ago. The failures attending this class of porcelain work are not due to the filling itself, as porcelain has a great many of the requirements of an "ideal" filling. It has the possibility of shading. It can restore the contour. The texture is sufficiently strong. It is easy of manipulation. It does not disintegrate or change in form by saliva. It is a poor conductor of heat or cold. It fills the bill for nervous patients and invalids, where the working of gold is next to impossible. It is particularly adapted for soft teeth where another dentist has failed with gold, and you come to the rescue with porcelain.

After this plea for porcelain inlay, wherein lies its weakness? It is in the material which connects the porcelain to the tooth; the "cements" are at fault. I was pleased to see the subject of cements brought up before the National Dental Association last August in a paper read by Dr. Wedelstaedt, in which he showed by demonstrations and his investigations that the cements for which we pay large prices are a miserable failure for their intended purpose. This is a subject that requires attention both by the manufacturers and the scientific workers along this line.

Our amalgam work in the mouth was in a similar state, our failures staring us in the face every day—we gullible dentists paying handsome prices for alloys giving no better, if as good, results as the "homespun" of forty years ago. We must thank such scientific men as Dr. Black for their persistent efforts, as in his experiments and agitation, for the improved alloy to be had to-day.

True enough, cements under different environments and conditions give different results. I have seen large cements fillings on the morsal surfaces of molars doing service for fifteen or twenty years without a perceptible disintegration, and seemingly as hard as flint. If we could rely on this kind of results in all mouths, the porcelain inlay would be the Mecca of fillings in conspicuous places, as you can get nearer the shading, transparency and density of the tooth than with any other material. The consistence of cements in mixing has no doubt a great deal to do with the durability. Thus of late I have been tempted to leave my old reliable "Justi" cement for inlays, because of its quick setting; whereas in using the Harvard the mixture can be given more body by a greater amount of powder, and the inlay can be pushed into its place without the granulation forming before the inlay is home, as very often happens with a quick-setting cement, when it is most annoying to be compelled to remove the inlay, scrape and wash out every particle of the cement, and try again.

With no better cements than we have I shall continue to put in porcelain inlays, because there are many who demand it, even if they are liable to require replacing in three, four, or five years. It has gained for me a reputation which I have to sustain, and those inlays which give way from time to time will no doubt be replaced in porcelain if in conspicuous places; but my experience has taught me *discrimination*, which should be a password to those ardent in first love.

"Permanent filling" is a definition in itself, and I am quite certain that we cannot as yet put porcelain inlays in that class, as they are limited by the success of the cement, which is itself classed as temporary.

In reviewing my record books on inlay work as food for this paper, I find the cavities on the labial surfaces of the incisors and cuspids, and also those at the gum-margin, are in the best state of preservation. The cements in the majority of cases have dissolved out more or less, but this does not seem detrimental to the tooth. In approximal cavities where the joint is at the gum-margin, the cements dissolve more rapidly, and recurrence of decay is sure to follow. The best results of any inlay are found in those which were done by the "How" system of trephining a cavity, and grinding the inlay in revolving motion. But this inlay, which is absolutely perfect as inlays go, has allowed the cement to dissolve out, and in some cases a slight dark line is shown by the infiltration of the saliva.

I have corners, contours, tips and cusps in all stages; some doing only fairly well, others better—depending entirely, of course, on the action of the saliva on the cements. I must say, however, after seeing some of the inlays that were put in eight and ten years ago still preserving the tooth and looking well, it is encouraging enough to continue the inlay process where properly indicated, in hopes of an insoluble cement being discovered. At any rate, it is well that one should be up to date and able to cope with the different requirements of the public, satisfying himself that his efforts are equal to the position he holds in the mind of the patient, whose interest should be paramount to every other consideration, and who should be given accurate information as to the applicability of the porcelain to his case in respect to appearance and durability as compared with gold and other fillings.

Will it last as long as gold? This question is invariably asked, and is a sticker when the cavity indicates porcelain and you have made a plea for it, your inclination at the same time being toward gold as the paragon filling for durability.

Some weeks ago I received a telegram from a lady (residing in a city in this State) asking an appointment to have replaced a large contour of porcelain which I had made some five or six years ago. On examination I found the enamel of the tooth badly broken, which made it necessary to remove the porcelain piece. Many questions were asked and answered as to the "pro" and "con" of porcelain and the relative merit of gold, the matter finally ending in the contour being made in platinum and gold as being the kind to meet the requirements to the satisfaction of both dentist and patient. It was most difficult to retain a porcelain filling in position, the large contour, on the mesial side of a central, having little or no depth to it, the pulp being intact. The lymphatic temperament of the patient gave a square occlusion to upper and lower incisors, which consequently had worn to a broad biting surface. There was also an approximal cavity on the distal surface, but not conspicuous. The porcelain was made for the mesial cavity. A groove was made in the remaining cutting-edge, and a thread-like platinum wire, baked into the inlay, ran along the floor of the cavity or groove on the cutting-edge and into the distal cavity, being built over with gold, and the cutting-edge shod to protect biting off the porcelain as well as the tooth.

The method I employ is to use a platinum matrix and high grade bodies, but the system has been explained in detail so often that no misstep could be taken by a beginner if he tried.

It seems to me there is only one way to get a proper matrix for an inlay; that is to burnish the metal, be it platinum or gold, into the cavity, which should have some depth, and not to apply merely a thin veneer, as some suggest. I see no advantage in trying to take a "perfect" impression of the cavity, whereby the matrix is made out of the mouth; it is a great loss of time. What better does one want than the tooth itself, with the true hard edges of the cavity to form the matrix against? You have gained nothing in going about trying to procure a duplicate of a cavity you already have at hand. One soon learns to remove a matrix without changing its form. One thing is certain, if there isn't space to get the matrix out freely you can not get the inlay in when made. Some workers in porcelain inlays advocate the filling of all undercuts with oxyphosphate, waiting until it hardens, and then make the cavity so the matrix can be got out very easily. In my working I have always relied on perfecting the edges of the matrix after having made a biscuit, which strengthens the matrix and allows it to be held and the edges burnished perfectly. In large contours I rather prefer to have an undercut at the gum margin, as it gives body to the fillings and everything helps as a retainer. With a little practice one is able to get the matrix down into a groove. With a platinum matrix it matters little if the metal should break through while burnishing, as the porcelain will bridge over the break and the inlay finish just as perfectly. It is sometimes necessary to retain a small corner on an incisor by forming a groove at the base of the cavity, and a staple of platinum wire is pushed through the bottom of the matrix and held in position with a little dampened body, and the whole withdrawn from the cavity and baked. If something of this kind is not done the corner is liable to be dislodged with a very slight strain.

It is quite common as a saving of time to make the inlay with one burnishing, the patient being dismissed and the inlay made in his absence at the leisure of the operator, who would require an excellent memory to remember the details of a contour, as so often the matrix does not indicate the shape of the lost part of the tooth, and only by trying into the cavity can one obtain the exactness that is necessary to get the required result.

Although the uses of porcelain and the furnace do not top here by any means, I feel that I must do so, before, completely exhausting those who have been good enough to listen to my feeble attempts.

